# Chi-miR-130b-3p regulates Inner Mongolia cashmere goat skin hair follicles in fetuses by targeting *Wnt family member 10A*

**DOI:** 10.1093/g3journal/jkaa023

**Published:** 2020-12-09

**Authors:** Zhihong Wu, Erhan Hai, Zhengyang Di, Rong Ma, Fangzheng Shang, Min Wang, Lili Liang, Youjun Rong, Jianfeng Pan, Rui Su, Zhiying Wang, Ruijun Wang, Yanjun Zhang, Jinquan Li

**Affiliations:** 1 College of Animal Science, Inner Mongolia Agricultural University, Hohhot 010018, Inner Mongolia, China; 2 Engineering Research Center for Goat Genetics and Breeding, Hohhot 010018, Inner Mongolia Autonomous Region, China; 3 Key Laboratory of Animal Genetics, Breeding and Reproduction, Hohhot 010018, Inner Mongolia Autonomous Region, China; 4 Key Laboratory of Mutton Sheep Genetics and Breeding, Ministry of Agriculture, Hohhot 010018, China

**Keywords:** chi-miR-130b-3p, cashmere goat, WNT10A, hair follicle, development, Wnt family, microRNA, fibroblast proliferation

## Abstract

The development of hair follicles (HFs) is dependent on interactions between epithelial cells and dermal fibroblasts, which may play an important role in maintaining the structure of HFs during their development and maturation. *Wnt family member 10* (*WNT10A*) is a hub gene during HF development and maturation that may regulate the proliferation of dermal fibroblasts and epithelial cells through microRNAs (miRNAs) and messenger RNAs (mRNAs) to maintain the structural stability of HFs. In the present study, we confirmed that *WNT10A* is the target gene of chi-miR-130b-3p by real-time quantitative PCR, western blotting, and a dual-luciferase reporter gene assay. We successfully cultured fetal epithelial cells and dermal fibroblasts using the tissue block attachment method, and Cell Counting Kit-8 (CCK8) results showed that chi-miR-130b-3p regulates epithelial cell and dermal fibroblast proliferation by targeting *WNT10A*.

## Introduction

Goats are renowned for their strong environmental adaptability and high resistance to stress. Based on production use, goats can be divided into dairy, cashmere, meat, skin, and other categories (Jiang *et al.* 2009). Inner Mongolia cashmere goat, an excellent local breed resulting from long-term natural selection and artificial breeding, is mainly produced in the western region of Inner Mongolia and is distributed in Erlang Mountain, Aerbasi, and Alxa Zuoqi (Jiang *et al.* 2009). The double coat of Inner Mongolian cashmere goats is composed of fluff and primary hair that grow from hair follicles (HFs) in the skin. HFs are composed of primary hair follicles (PHFs) and secondary hair follicles (SHFs). Skin forms a complete epidermal structure during the embryonic period from 45 to 55 days in Inner Mongolia cashmere goats, and PHFs of the fetal head, shoulder, and neck begin to form at 65 days (Wenjing *et al*. 2020). The PHF primordial body can be observed on the side of the body, and at 65 − 75 days, SHFs begin to appear in various parts of the fetus, generally growing from the epidermis near PHFs. Similar to PHFs, SHFs on the side of the body form later than in the other parts, and the SHF primordial body is not obvious until day 75. Some PHFs mature at 115 days and some SHFs mature at 135 days (Wenjing et al. 2020). The traits of HFs directly affect the yield and quality of fluff, but the traits of SHFs have no direct influence on the yield and quality of cashmere. Thus, studying the molecular regulatory mechanism governing HF growth and development in cashmere goat could be of significant economic value.

Most research on skin and HFs in cashmere goat has focused on changes in PHFs and SHFs during their growth, degeneration, and resting stages, and on the mechanisms of their related genes. The fetal development of PHFs and SHFs has been less well studied, and most studies on fetal skin and HF development have largely focused on the occurrence of PHFs and SHFs (fetal period 45–65 days). However, there have been no reports on the maintenance of HF structure and skin HF homeostasis by epithelial cells and dermal fibroblasts during the development and maturation of PHFs and SHFs (115–135 days). The stability of epithelial cells and dermal fibroblasts has been shown to be a key requirement for HFs to enter the hair cycle, and to maintain the normal HF structure in various mammals (Castilho *et al.* 2009).

MicroRNAs (miRNAs) are single-stranded small RNAs ∼22 nucleotides (nt) in length (Lagos-Quintana *et al.* 2001; Lau *et al.* 2001). They are from the short hairpin structure of miRNA precursors (pre-miRNAs) (Bartel 2004). MiRNAs were first discovered in the early 1990s (Lee *et al.* 1993; Wightman *et al.* 1993), and thousands of miRNAs have been found in animals and plants (Kozomara et al. 2019). MiRNAs influence several major biological pathways by regulating many protein-coding genes (Bartel 2018; Liu *et al.* 2018). Because gene regulation by miRNAs is ubiquitous, misregulation or abnormal expression of single miRNAs may have terrible consequences (Paul *et al.* 2018). In experiments on mice knockout, miRNA mature bodies were found to target the key enzymes dicer 1, ribonuclease III (Dicer) (Andl et al. 2006), drosha ribonuclease III (Drosha) (Yi *et al.* 2006), and DGCR8 microprocessor complex subunit (DGCR8) (Sohn et al. 2007; Yi *et al.* 2009), explaining their important role in the growth and development of HFs. Dicer knockout mice have poorly developed HFs and insufficient proliferation, and *Sonic Hedgehog* and *Notch Receptor 1*, key signaling factors in HF development, are lost at day 7 after birth, which results in the differentiation of the inner root sheath and hair stem, and HF mutations. At this stage, the dermal papillae of HFs are inverted and form unusual structures within the basal epidermis, rather than normal hair stems, and HFs lack stem cell markers and gradually degenerate (Andl et al. 2006). In pluripotent progenitor *Dicer1* knockout embryonic skin cells, cell division and differentiation were not significantly impaired within 1 week of miRNA expression loss, and there was no significant increase in apoptosis in the inter follicular epidermis, but dermal papilla cells that appeared after differentiation did not invade and were turned outward, which disrupted the normal structure of epidermal tissue (Yi *et al.* 2006). *DGCR8* is the main protein of the *Drosha* complex. In the process of producing pre-miRNAs, *DGCR8* plays a role in recruiting and guiding *Drosha* to cut in the correct position of pre-miRNAs (Sohn *et al.* 2007). Changes in embryonic skin tissue lacking *DGCR8* are highly consistent with skin changes observed in the absence of Dicer (Yi *et al.* 2009). Although there is increasing evidence that miRNAs play an important role in HFs, there are few reports on HFs in cashmere goats.

Using weighted gene co-expression network analysis, our research group showed that *WNT10A* is a hub gene controlling skin development and maturation during the fetal stage in Inner Mongolia cashmere goat (Zhihong *et al*. 2020). We speculate that Wnt10a may support the normal development of HFs by maintaining the proliferation of epithelial cells and dermal fibroblasts during this stage, and overexpression of *WNT10A* at this stage may be achieved through a mechanism involving miRNA–mRNA interactions.

## Materials and methods

### Animals

Samples were obtained from Inner Mongolia Jinlai Animal Husbandry Technology Co., Ltd. (Hohhot, Inner Mongolia). The environment of the cashmere goat farm meets the relevant requirements of the experimental facilities in the Chinese national standard “Experimental Animal Environment and Facilities” (GB14925-2010). Health status, pathogenic microorganism infections, and zoonotic infections were monitored to ensure animal safety. Mating of the experimental animals was completed in the natural state of estrus.

According to the breeding records, we performed cesarean section on ewes at different gestational periods. During the operation, general anesthetic was used to relieve pain, and ewes were monitored and nursed. All operating personnel were qualified veterinarians possessing relevant certificates and more than 3 years of clinical experience. We collected lateral skin tissue samples from goat fetuses at seven periods (three fetuses from each period), using diethyl pyrocarbonate water to clean embryos after cesarean section. Skin samples (1 cm^2^) were rapidly collected using a sterile, enzyme-free disposable scalpel and forceps, placed in frozen storage tubes, numbered, quickly frozen in liquid nitrogen, and stored at −80°C. The sampling process was conducted in strict accordance with animal welfare requirements.

All fetal skin samples were collected in accordance with the International Guiding Principles for Biomedical Research Involving Animals and approved by the Special Committee on Scientific Research and Academic Ethics of Inner Mongolia Agricultural University, responsible for the approval of biomedical research ethics of Inner Mongolia Agricultural University (Approval No. [2020] 056). No specific permissions were required for these activities, and no endangered or protected species were involved.

### Real-time quantitative PCR

Our previous bioinformatics research revealed a potential regulatory relationship between chi-miR-130b-3p and *WNT10A* in Inner Mongolia Cashmere goat (Zhihong *et al.* Unpublished). We therefore used real-time quantitative PCR (RT-qPCR) to verify whether the gene expression levels of chi-miR-130b-3p and *WNT10A* were negatively correlated at seven fetal stages (45, 55, 65, 75, 95, 115, and 135 days). Total RNA was isolated from skin samples at the seven fetal stages using TRIzol (Invitrogen 10296-010, Carlsbad, CA, USA) and reverse-transcribed into cDNA using a PrimeScript Reagent Kit (Takara Biomedical Technology Co., Ltd, Beijing Municipality, China). RT-qPCR was performed using a Fluorescence Quantitative PCR Kit (Takara), and mRNA expression levels were calculated using the 2^−ΔΔCT^ method (Schmittgen and Livak 2008). The Spearman correlation coefficient (Rs) was used for normalization.

### Dual-luciferase reporter gene assay

Primers for amplifying *WNT10A* and the *WNT10A* 3ʹ-untranslated region (UTR) were designed based on the gene sequence in GenBank, and the 3ʹ-UTR sequence of the gene was amplified by PCR using Cashmere goat genomic DNA as template. PCR products were cloned into the pSI-checK2 dual-luciferase reporter gene vector (Hanheng Biotechnology Co., Ltd., Shanghai, Municipality, China) to construct the wild-type plasmid. The target sequence of chi-miR-130b-3p in the *WNT10A* gene was mutated to construct a mutant plasmid. Finally, expression of the luciferase reporter was measured, and the target site of miRNA in the transfected 3ʹ-UTR was analyzed. The plasmid was synthesized by Shanghai Hanheng Biotechnology Co., Ltd.

### Culture and identification of dermal fibroblasts and epithelial cells at different fetal stages

Fetal dermal fibroblasts and epithelial cells were cultured by the tissue block adhesion method and identified by morphological observation and immunofluorescence.

### Construction of lentivirus-mediated chi-miR-130b-3p interference and overexpression plasmids

This part of the study was conducted by Shanghai Hanheng Biotechnology Co., Ltd. Animals were divided into negative control (NC), HBLV-chi-miR-130b-3p-sponge-ZsGreen-PURO, and HBLV-chi-miR-130b-3p-ZsGreen-PURO groups.

### Construction of HBLV-chi-miR-130b-3p-sponge-ZsGreen-PURO and HBLV-chi-miR-130b-3p-ZsGreen-PURO dermal fibroblast and epithelial cell lines

The third generation of epithelial cells and dermal fibroblasts was digested with trypsin and inoculated into 24-well plates. When cells reached 50 − 60% confluence, the cell medium was discarded and replaced with 1/2 the volume of fresh culture medium containing lentivirus. After 4 h of infection in a 37°C incubator with 5% CO_2_, the culture medium was replenished to the normal volume. On the second day after infection, the culture medium containing the virus was discarded and replaced with fresh culture medium without the virus. At 72 h after infection, the infection efficiency was ∼80%, and when fluorescence was high, it was observed using a fluorescence microscope (Hanheng, China). Puromycin (PURO) was added for resistance screening to kill cells that had been successfully transfected, and a 1/2 volume of PURO was added for culture maintenance.

### Functional analysis of chi-miR-130b-3p in dermal fibroblasts and epithelial cells

Total RNA was isolated from epithelial cells and dermal fibroblasts using TRIzol and reverse-transcribed into cDNA using a PrimeScript reagent Kit (Takara). RT-qPCR was performed using a Fluorescence Quantitative PCR Kit (Takara), and mRNA expression levels were calculated using the 2^−ΔΔCT^ method. Western blotting was performed to detect WNT10A protein in dermal fibroblasts and epithelial cells transfected with chi-miR-130b-3p. Briefly, after the cell line was successfully constructed, total protein was extracted and the protein concentration was determined by the BCA protein assay method. Proteins were separated by sodium dodecyl sulfate-polyacrylamide gel electrophoresis (SDS-PAGE), transferred to a membrane, and incubated with WNT10A primary antibody (Affinity Biosciences LTD, Jiangsu Province, China) overnight at 4°C. After washing, the membrane was incubated with different concentrations of secondary antibody (Boster Bioengineering Co., Ltd., Wuhan, China) at room temperature for 1 h. Enhanced chemiluminescence (ECL) reagent was used for visualization with a gel imaging analysis system. The exposure time was adjusted for photos.

### Cell proliferation assay

Cell proliferation was measured by the CCK8 assay (TIANGEN BIOTECH CO., Ltd., Beijing Municipality, China) according to the manufacturer’s instructions. At 72 h after transfection, cells were seeded in 96-well plates and the optical density of each well was determined at 490 nm.

### Data availability

All data supporting the conclusions of this work are included in the article.

## Results

### Chi-miR-130b-3p targets and regulates WNT10A

RT-qPCR results showed that *WNT10A* and chi-miR-130b-3p were both expressed at all seven cashmere goat fetal stages, and the data revealed opposite expression trends. There was a strongly negative Spearman’s correlation coefficient (Rs = −0.82) between chi-miR-130b-3p and *WNT10A* expression at all seven stages.

**Figure 1:**
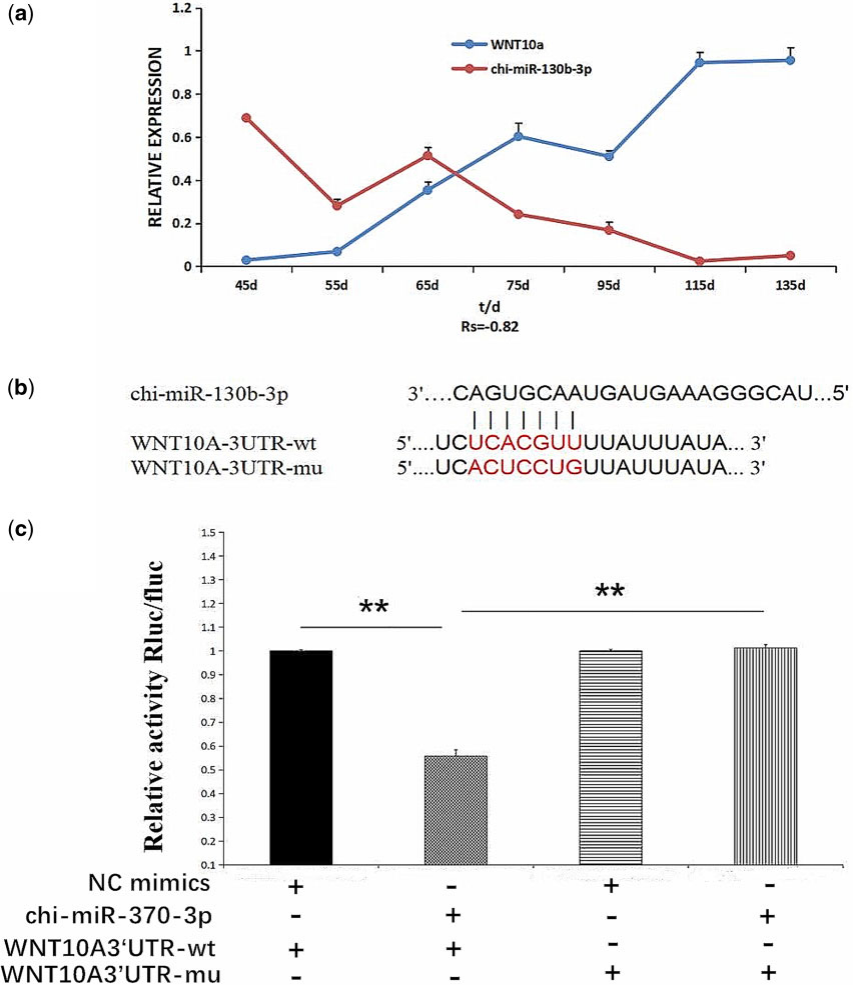
Verification of the targeting relationship between chi-miR-130b-3p and *WNT10A* in Inner Mongolia cashmere Goat. (A) Relative expression of C chi-miR-130b-3p and *WNT10A* at different fetal periods. The correlation between the two variables is expressed by the correlation coefficient Rs (Rs = −0.82, indicating a significant negative correlation). (B) Chi-miR-130b-3p and WNT10A-3ʹ-UTR binding sites and mutation sites. (C) Verification of the interaction between chi-miR-130b-3p and WNT10A-3ʹ-UTR detected by a dual-luciferase reporter gene assay (**P < 0.01). Results in A and C are expressed as mean ± standard error of the mean (SEM).

To further investigate the relationship between chi-miR-130b-3p and WNT10A, we cloned the WNT10A-3ʹ-UTR fragment into the pSI-checK2 dual-luciferase reporter gene vector, and based on prediction of the WNT10A-3ʹ-UTR and miRNA-targeted binding sites, potential binding sites between chi-miR-130b-3p and WNT10A were determined (Figure 1B). WNT10A was confirmed to be a potential target gene of chi-miR-130b-3p. Finally, chi-miR-130b-3p significantly downregulated the expression of the wild-type WNT10A-3ʹ-UTR according to the results of the dual-luciferase reporter gene assay system, indicating binding between the two molecules, and this downregulation effect disappeared after mutation, indicating that the mutation was successful (Figure 1C).

### Culturing of Inner Mongolia cashmere goat dermal fibroblasts and epithelial cells

**Figure 2:**
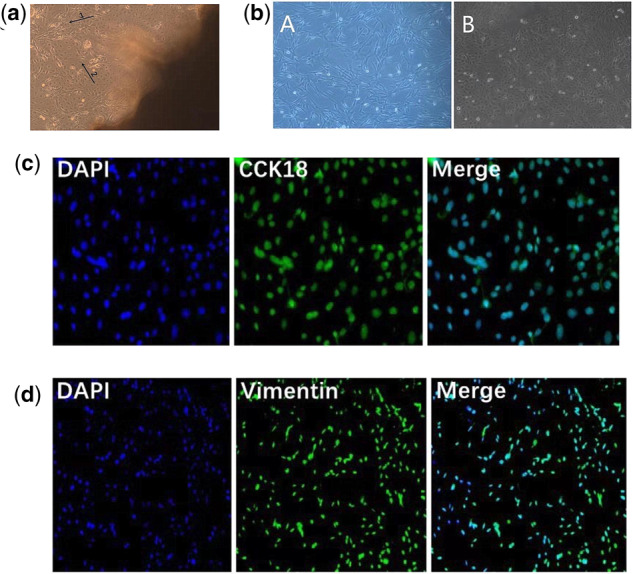
Culture and identification of fetal skin epithelial cells and dermal fibroblasts of Inner Mongolia cashmere goat. (A) Primary dermal fibroblasts and epithelial cells. Arrows 1 and 2 indicate fibroblasts and epithelial cells, respectively. (B) Dermal fibroblasts and epithelial cells. (C) Immunofluorescence identification of epithelial cells. (D) Immunofluorescence identification of dermal fibroblasts.

Primary cells were successfully isolated from the Inner Mongolia Cashmere goat fetal skin samples by the tissue block attachment method (Figure 2A) and purified by trypsin digestion to obtain fetal fibroblasts and epithelial cells (Figure 2B). Keratin 18 (CCK18) is a unique skeleton protein of epithelial cells. Keratin 18 was confirmed to be present on the epithelial cell membrane by immunofluorescence, and nuclei were successfully stained with 4ʹ,6-diamidino-2-phenylindole (DAPI), indicating that goat fetal skin epithelial cells were successfully cultured (Figure 2C).

Vimentin is a specific protein on the surface of the dermal fibroblast membrane. Vimentin was confirmed to be present on the dermal fibroblast membrane by immunofluorescence, and nuclei were successfully stained with DAPI, indicating that goat fetal dermal fibroblasts were successfully cultured (Figure 2D).

### Construction of HBLV-chi-miR-130b-3p-sponge-ZsGreen-PURO and HBLV-chi-miR-130b-3p-ZsGreen-PURO dermal fibroblast and epithelial cell lines

**Figure 3:**
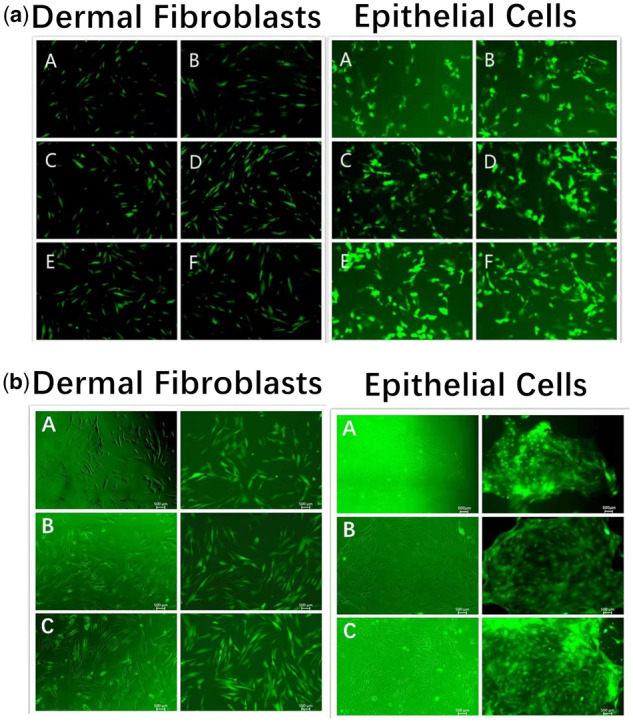
Construction of HBLV-chi-miR-130b-3p-sponge-ZsGreen-PURO and HBLV-chi-miR-130b-3p-ZsGreen-PURO dermal fibroblast and epithelial cell lines. (A) Determination of multiplicity of infection (MOI) values for epithelial cells and dermal fibroblasts. a, b, d, e, and f are fetal epithelial cells transfected with lentiviral empty vector at MOI values of 1, 2, 3, 4, 5, and 6, respectively. (B) Construction of HBLV-chi-miR-130b-3p-sponge-ZsGreen-PURO and HBLV-chi-miR-130b-3p-ZsGreen-PURO dermal fibroblast and epithelial cell lines (a–c are NC, HBLV-chi-miR-130b-3p-sponge-ZsGreen-PURO group, and HBLV-chi-miR-130b-3p-ZsGreen-PURO groups, respectively).

First, we determined that the optimal titers of lentivirus-infected dermal fibroblasts and epithelial cells were 4 and 5 TU number/cell, respectively (Figure 3A). After PURO resistance screening, fluorescence microscopy showed that resistance screening was successful; both dermal fibroblasts (Figure 3B) and epithelial cells (Figure 3B) exhibited strong green fluorescence with a uniform distribution. Thus, both HBLV-chi-miR-130b-3p-sponge-ZsGreen-PURO and HBLV-chi-miR-130b-3p-ZsGreen-PURO dermal fibroblast and epithelial cell lines were successfully constructed.

### Functions of chi-miR-130b-3p in fetal fibroblasts and epithelial cells

**Figure 4:**
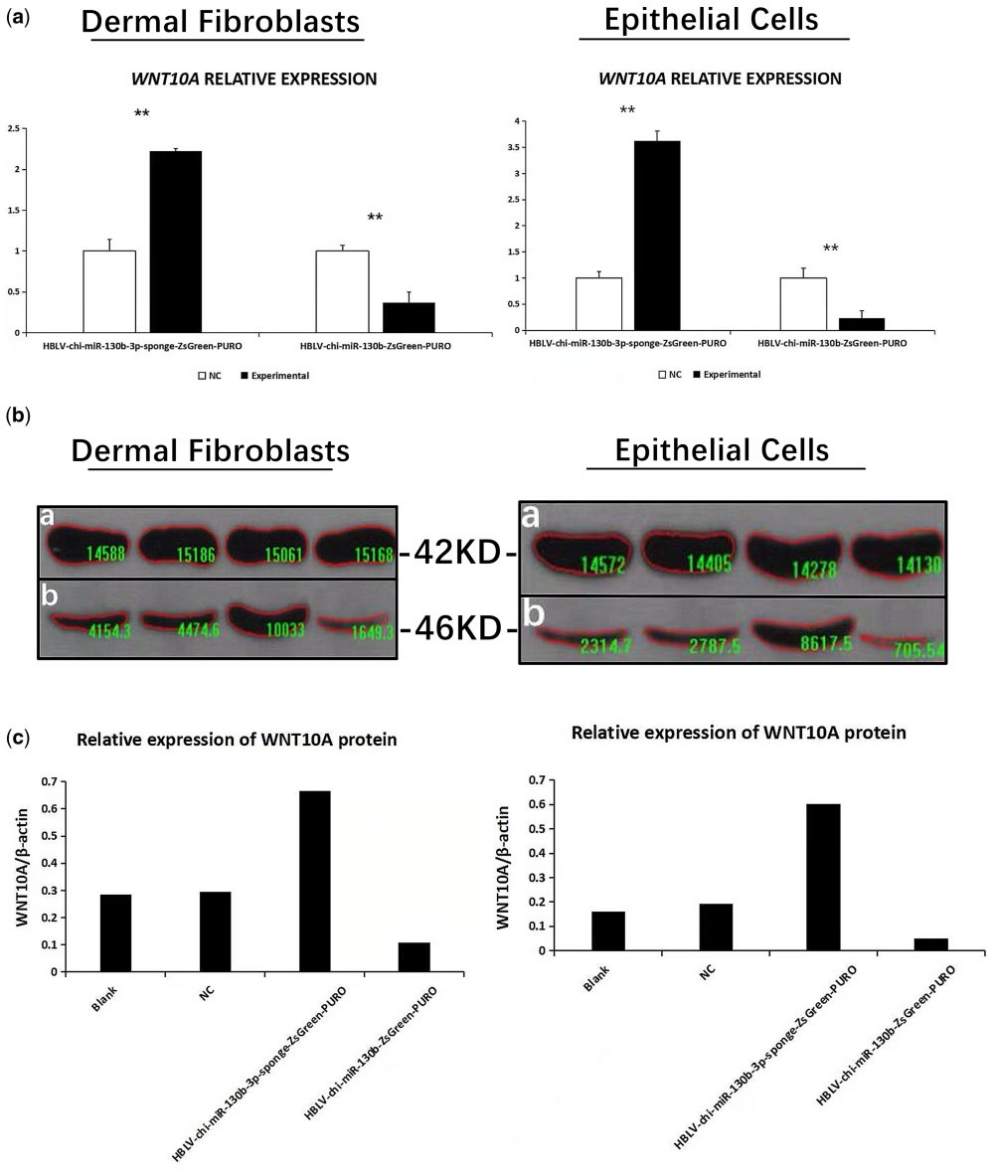
Functional verification of chi-miR-130b-3p in the post-transcriptional regulation of *WNT10A*. (A) RT-qPCR results (**P <* *0.01; mean ± SEM). (B) Expression of β-actin and WNT10A proteins in each cell line. (C) Relative protein expression of WNT10A in different cell lines.

**Figure 5:**
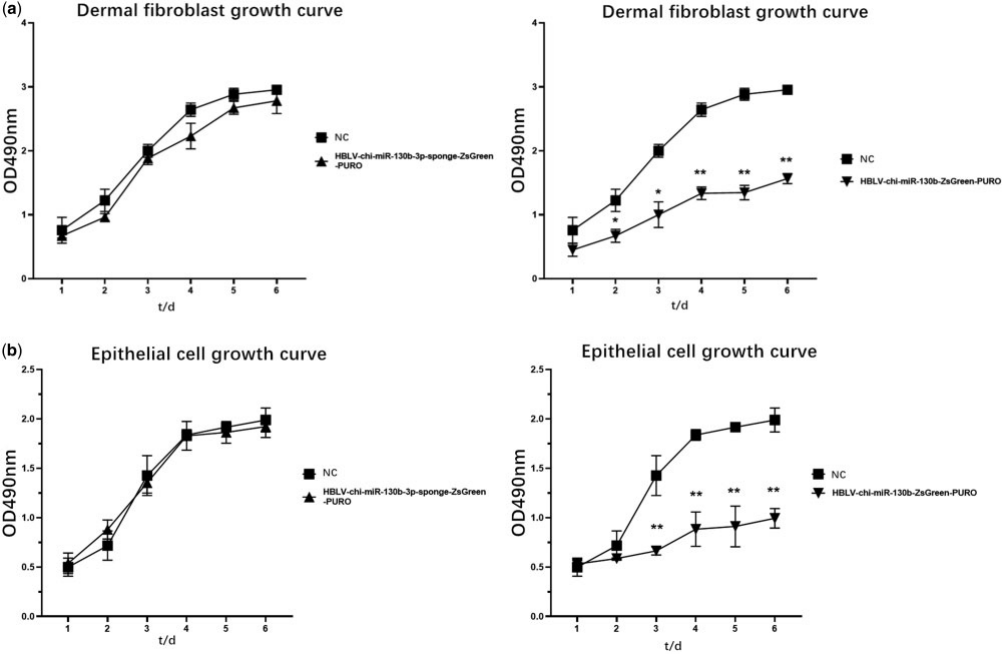
Cell growth curve for each cell line (*P <* *0.05, **P < 0.01; mean ± SEM).

RT-qPCR results showed that after transfection of dermal fibroblasts (Figure 4A, Table 1), expression of *WNT10A* in the HBLV-chi-miR-130b-3p-sponge-ZsGreen-PURO group was 2.2245 times higher than that in the NC group (*P *<* *0.01). Meanwhile, expression of *WNT10A* in the HBLV-chi-miR-130b-3p-ZsGreen-PURO group was 0.368 times higher than that in the NC group (*P *<* *0.01). *WNT10A* can promote the proliferation of dermal fibroblasts and epithelial cells (Figure 5).

**Table 1:** Relative expression of *WNT10A* in different experimental groups of dermal fibroblasts from Inner Mongolia Cashmere goat

	NC	Experimental	*P*-value
HBLV-chi-miR-130b-3p-sponge-ZsGreen-PURO	1 (±0.14)	2.2245 (±0.032)	<0.01
HBLV-chi-miR-130b-ZsGreen-PURO	1 (±0.0717)	0.368 (0.1322)	<0.01

After transfection of epithelial cells (Figure 4A, Table 2), expression of *WNT10A* in the HBLV-chi-miR-130b-3p-sponge-ZsGreen-PURO group was 3.6206 times higher than that in NC group (*P *<* *0.01). Expression of WNT10A in the HBLV-chi-miR-130b-3p-ZsGreen-PURO group was 0.232 times higher than that in the NC group (*P *<* *0.01).

**Table 2:** Relative expression of *WNT10A* in different experimental groups of fetal epithelial cells from Inner Mongolia Cashmere goat

	NC	Experimental	*P-*value
HBLV-chi-miR-130b-3p-sponge-ZsGreen-PURO	1 (±0.123)	3.62 (±0.1952)	<0.01
HBLV-chi-miR-130b-ZsGreen-PURO	1 (±0.19)	0.232 (±0.1443)	<0.01

Western blotting results analyzed by Image-Pro Plus yielded gray values for WNT10A and β-actin for each treatment group of fetal fibroblasts and epithelial cells (Figure 4B), and the relative expression of WNT10A protein in each experimental group of fetal fibroblasts (Figure 4C) and fetal epithelial cells (Figure 4C).

In fetal fibroblasts, WNT10A/β-actin values for blank, NC, HBLV-chi-miR-130b-3p-sponge-ZsGreen-PURO, and HBLV-chi-miR-130b-3p-ZsGreen-PURO groups were 0.285, 0.295, 0.666, and 0.109, respectively (Table 3).

**Table 3:** Relative expression of *WNT10A* protein

	Blank	NC	HBLV-chi-miR-130b-3p-sponge-ZsGreen-PURO	HBLV-chi-miR-130b-ZsGreen-PURO
Dermal fibroblasts	0.285	0.295	0.667	0.109
Epithelial cells	0.159	0.194	0.604	0.050

In fetal epithelial cells, WNT10A/β-actin values for blank, NC, HBLV-chi-miR-130b-3p-sponge-ZsGreen-PURO, and HBLV-chi-miR-130b-3p-ZsGreen-PURO groups were 0.285, 0.295, 0.666, and 0.109, respectively. These results confirm that chi-mir-130b-3p negatively regulates the WNT10A gene during HF development in goat fetal skin (Table 3).

## Discussion

The induction and formation of HFs are regulated by interactions between specific dermal fibroblasts and adjacent epithelial cells. During the process of HF growth, communication between dermal papilla cells and surrounding epithelial cells coordinates the formation of hair stems (Hawkshaw *et al.* 2020). In the present study, *WNT10A* was found to assist the homeostasis of epithelial cells and dermal fibroblasts through regulation by chi-miR-130b-3p.

Epithelial cells and dermal fibroblasts are key players in HF development, and these cells maintain the normal structure and functions of HFs during maturation. For example, slow proliferation of epithelial cells results in the epidermis aging, eventually leading to hair loss (Castilho *et al.* 2009). When the mitosis of epithelial cells is inhibited, dermal papilla cells lose the ability to induce HF regeneration. They fail to differentiate into HFs, but can differentiate into epidermal cells. Importantly, HF stem cells lose the ability to proliferate, which also blocks the transition of HFs from the resting stage to the growth stage (Huang *et al.* 2017). In mice in which the ability to form epithelial cells is lost, the epidermal barrier is damaged, and eventually, HFs will enter into the catagen phase (Hamanaka *et al.* 2013). It is generally believed that epithelial cells cannot form a placode again after HF maturation. However, recent studies have shown that epithelial cells in the mature stage of HFs can also form HF-like structures under the stimulation of dermal papilla cells (Ehama *et al.* 2007).

Dermal fibroblasts are one of the main components of the skin and play a key role in the process of skin aging. The replicative aging of dermal fibroblasts may lead to skin aging, HF atrophy, poor wound healing, skin diseases, and even cancer (Meng *et al.* 2018). In the process of HF circulation, dermal accumulation of fibroblasts is the “energy source” of HFs at each growth stage (Marsh *et al.* 2018).

Maintaining genomic integrity is essential to the stability of the tissue environment. *Hes family bHLH transcription factor 1* (*Hes1*) is a key transcription factor downstream of the Notch signaling pathway. Knocking out *Hes1* in epithelial cells slows hair growth, and the loss of *Hes1* delays the activation and shortens the growth period of secondary HFs, which leads to the shortening of hair stems of secondary HFs (Suen *et al.* 2020). *TFAM (Eko)* is an important maintenance factor in the mitochondria of epithelial cells, and a lack of *TFAM (Eko)* in mice can lead to epithelial cell apoptosis and reduced proliferation, resulting in HFs in the growth phase entering the resting phase earlier (Kloepper *et al.* 2015). *Podoplanin* (*PDPN*) is a glycoprotein that is highly expressed in epithelial cells. The adhesion of epithelial cells lacking *PDPN* is reduced, which eventually leads to the shortening of hair circulation (Yoon *et al.* 2019).

In conclusion, regulation of *WNT10A* expression by chi-miR-130b-3p may play an important role in the development and maturation of HFs in Inner Mongolia cashmere goat.

## Funding

This research was funded by the National Natural Science Foundation of China (31860627) and the Plan Project of Science and Technology in Inner Mongolian (2019GG243).

*Conflicts of interest*: The authors declare there is no conflicts of interest regarding the publication of this paper.
